# A Case of Long-Term Management of Oral Function in a Child with Dyskeratosis Congenita

**DOI:** 10.3390/diagnostics16091358

**Published:** 2026-04-30

**Authors:** Erika Aoki, Keigo Kubota, Kazuto Hoshi

**Affiliations:** Department of Oral and Maxillofacial Surgery and Orthodontics, The University of Tokyo Hospital, Tokyo 113-8655, Japan

**Keywords:** dyskeratosis congenita, telomere biology disorder, pediatric dentistry, short root anomaly, chemotherapy, oral management, panoramic radiography

## Abstract

**Background and Clinical Significance**: Dyskeratosis congenita (DC) is a rare inherited disorder classified as a telomere biology disorder and characterized by multisystem involvement, including bone marrow failure and mucocutaneous abnormalities. Oral manifestations such as leukoplakia, increased susceptibility to infection, and abnormal dental development have been reported; however, detailed descriptions of long-term oral functional management in pediatric patients remain limited. This report aims to describe the longitudinal oral management and imaging-based assessment of dental development in a child with DC and to discuss the clinical implications in the context of existing literature. **Case Presentation**: A female patient diagnosed with dyskeratosis congenita due to a heterozygous TINF2 mutation was followed from early childhood. She underwent hematopoietic stem-cell transplantation at five years of age and later required lung and liver transplantation. Long-term oral management included regular professional oral care, preventive strategies, and periodic imaging evaluation. Panoramic radiographs obtained over several years demonstrated generalized delayed eruption and incomplete root formation relative to chronological age, with apparent early arrest of root elongation. **Discussion**: This case highlights the potential association between telomere dysfunction, intensive systemic therapy in early childhood, and arrested odontogenesis. These findings suggest a possible association between telomere dysfunction, early intensive systemic therapy, and impaired root formation. Despite severe systemic disease, continuous preventive oral care and imaging-based monitoring were effective in maintaining oral health and detecting mucosal changes. **Conclusions**: Long-term preventive oral management combined with noninvasive imaging assessment may play an important role in preserving oral function and monitoring dental development in pediatric patients with dyskeratosis congenita. This case adds to the limited literature on longitudinal oral outcomes in this rare disorder.

## 1. Introduction

Dyskeratosis congenita (DC) is a rare inherited disorder within the spectrum of telomere biology disorders (TBDs), caused by pathogenic germline variants in genes involved in telomere maintenance (e.g., DKC1, TERC, TERT, RTEL1, and TINF2) and characterized by very short telomeres and premature cellular senescence [1,2,3,4]. Clinically, DC has been classically described as a mucocutaneous triad of nail dystrophy, reticular skin pigmentation, and oral leukoplakia; however, phenotypes are heterogeneous and may include bone marrow failure, immunodeficiency, pulmonary and liver disease, and other multisystem complications [2,3].

Bone marrow failure is a major contributor to morbidity and mortality in DC/TBDs, and hematopoietic stem-cell transplantation (HSCT) is often required in childhood [2,3]. Even with reduced-intensity conditioning, treatment-related toxicities and long-term sequelae remain important because telomere dysfunction impairs stem-cell reserves and tissue repair capacity, potentially amplifying vulnerability to chemotherapy and radiation exposures [2,3].

DC/TBDs show substantial phenotypic heterogeneity and overlap with other inherited bone marrow failure syndromes, which can complicate clinical recognition and diagnosis [5,6]. Measurement of leukocyte telomere length is commonly used to support diagnosis and to inform risk stratification [7]. Beyond marrow failure, vascular and gastrointestinal complications—including bleeding—have also been described in TBDs [8,9], and somatic genetic rescue may modify hematologic phenotypes over time in some Mendelian disorders [10].

Oral involvement in DC/TBDs is clinically significant and extends beyond leukoplakia. Reported findings include mucosal atrophy and fragility, dental caries and periodontal inflammation, enamel and tooth-shape anomalies, taurodontism, and developmental disturbances of dental root formation, including blunted or short roots [3,11,12,13,14,15,16]. Because root development continues for years after eruption, insults during odontogenesis can result in permanent root shortening, compromised tooth stability, and long-term functional limitations.

Despite increasing recognition of oral manifestations in DC/TBDs, detailed longitudinal reports integrating continuous oral management with serial radiographic evaluation in pediatric patients remain limited [13]. Consequently, the natural history and timing of root developmental arrest in DC—particularly in the context of intensive systemic therapy—are incompletely defined.

In this context, longitudinal imaging-based assessment (e.g., serial panoramic radiographs) provides objective documentation of dental development over time and may help clinicians distinguish developmental disturbances related to the underlying telomere biology disorder from treatment-related sequelae [17,18,19,20,21,22,23,24]. By presenting long-term oral functional management with serial radiographic findings in a child with DC, this report aims to contextualize the clinical course against the existing literature and to highlight practical considerations for preventive oral care in medically complex children [3,13,24].

## 2. Case Presentation

### 2.1. Patient Information and Medical History

The patient was a female child who first visited the Department of Oral and Maxillofacial Surgery and Orthodontics at five years of age for intraoral evaluation prior to hematopoietic stem-cell transplantation. She had been born without perinatal complications. At the age of three years, she developed a fever and bleeding tendency associated with pancytopenia and was diagnosed with refractory thrombocytopenia. Subsequent genetic analysis revealed a heterozygous TINF2 R282H mutation, confirming the diagnosis of dyskeratosis congenita.

Her medical history was notable for multiple systemic complications, including rickets with bilateral enlargement of the epiphyseal lines of the lower extremities, bilateral pulmonary arteriovenous malformations, disseminated herpes zoster infection, interstitial pneumonia, and multiple brain abscesses. These findings were consistent with the multisystem involvement typical of severe DC.

In January 2015, at the age of five years, the patient underwent allogeneic HSCT using a reduced-intensity conditioning regimen consisting of fludarabine (100 mg/m^2^), cyclophosphamide (3000 mg/m^2^), and antithymocyte globulin (5 mg). Although hematological engraftment was achieved, she experienced several infectious and organ-related complications during subsequent years.

### 2.2. Clinical Findings

**Figure 1:**
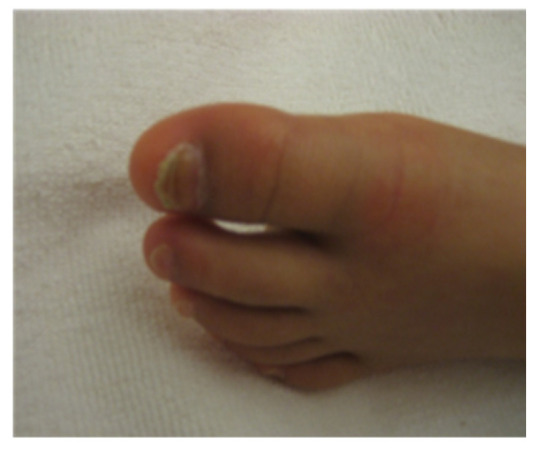
Nail atrophy observed in the patient, which is one of the characteristic features of dyskeratosis congenita.

**Figure 2:**
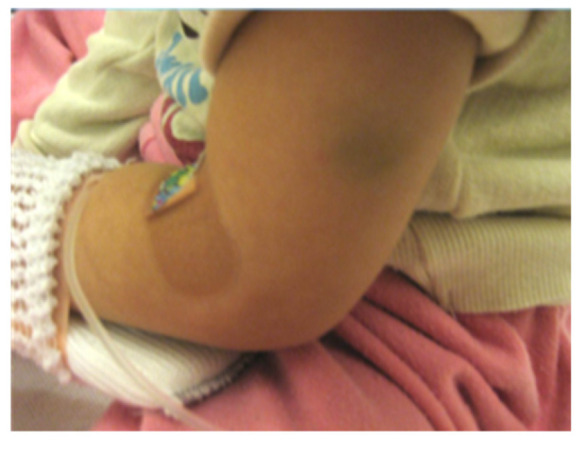
Dry skin and subcutaneous hemorrhagic scars observed on physical examination.

**Figure 3:**
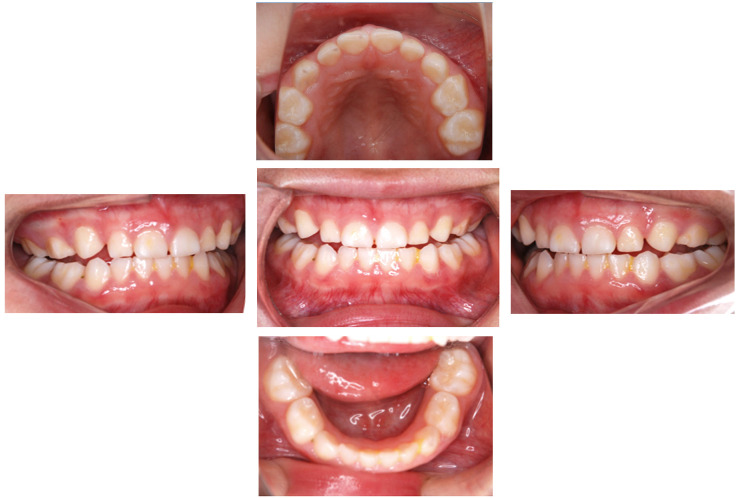
Intraoral findings at the initial visit showing plaque accumulation on the anterior teeth and gingival inflammation without dental caries.

Extraoral examination revealed nail atrophy, dry skin, and subcutaneous hemorrhagic scars, which were consistent with the characteristic mucocutaneous features of DC (Figure 1 and Figure 2). Intraoral examination at the initial visit showed primary dentition appropriate for age (Figure 3). No dental caries were detected; however, plaque accumulation was evident on the anterior teeth, accompanied by gingival inflammation. Oral mucositis and a well-demarcated white lesion on the dorsal surface of the tongue were also observed, raising concern for leukoplakia, a known manifestation of DC.

### 2.3. Imaging Findings and Dental Development

**Figure 4:**
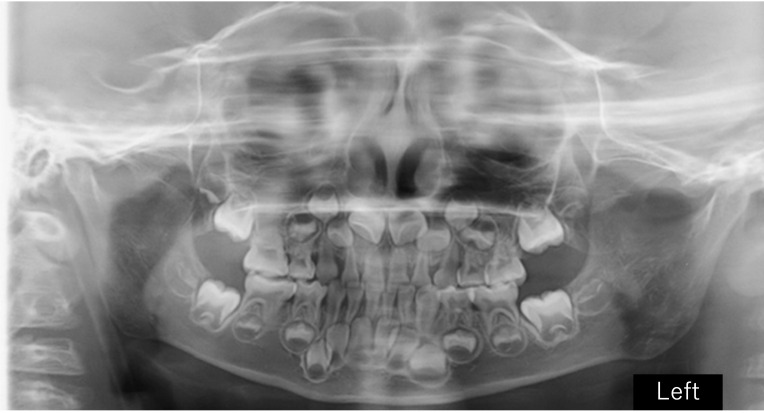
Panoramic radiograph at 5 years of age demonstrating primary dentition with age-appropriate development. No abnormalities in the number of developing permanent tooth germs were observed.

**Figure 5:**
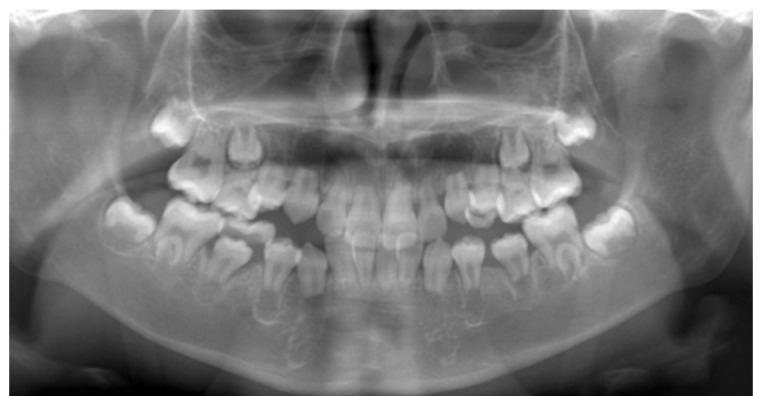
Panoramic radiograph at the age of 7 years showing mixed dentition. No abnormalities were observed in the number of permanent teeth. Generalized short-root morphology was present throughout the dentition, without variation by tooth type or anatomical location.

**Figure 6:**
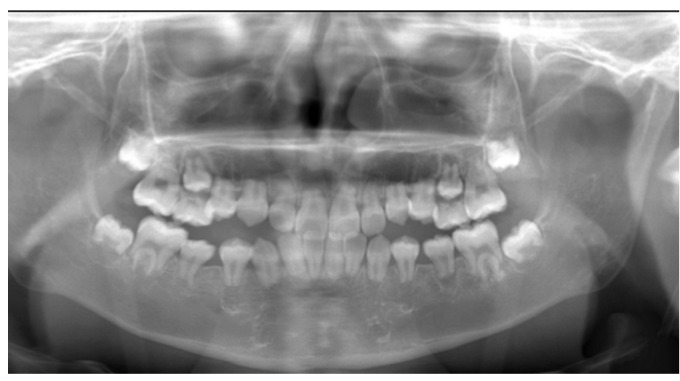
Panoramic radiograph at nine years of age demonstrating exfoliation of the right mandibular second primary molar and the left maxillary first primary molar, without additional root growth compared with the 7-year image.

Panoramic radiographs were obtained periodically between the ages of five and eleven years as part of routine dental evaluation and treatment planning. At the initial visit, panoramic radiographic findings at five years of age showed no abnormalities in the number of permanent teeth, and crown formation had already begun. The findings were consistent with normal development for the patient’s chronological age (Figure 4). Between the ages of five and ten years, the exfoliation of primary molars and the eruption of permanent premolars were observed bilaterally (Figure 5 and Figure 6). However, root elongation of the erupting permanent teeth was markedly limited.

**Figure 7:**
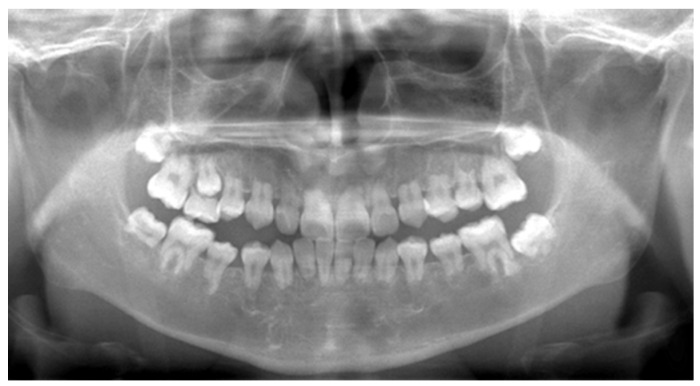
Panoramic radiograph at the age of 10 years showing the exfoliation of the left maxillary second primary molar and the eruption of the left maxillary second premolar, with no additional root development observed throughout the dentition.

**Figure 8:**
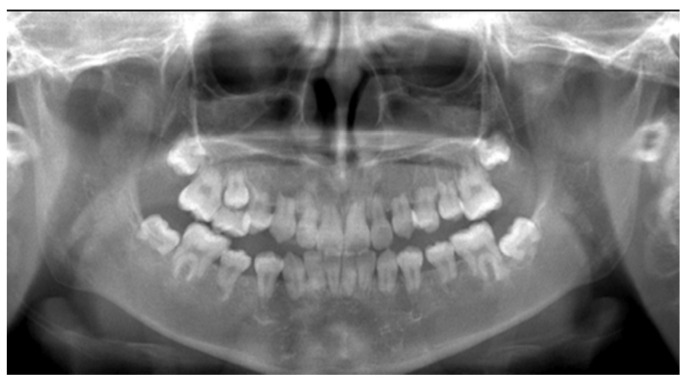
Panoramic radiograph at the age of 11 years showing no additional exfoliation of primary teeth or further root development compared with the radiograph obtained at 10 years of age.

Between nine and ten years of age, exfoliation was limited to the left maxillary second primary molar, and no additional permanent tooth eruption or root growth was noted. The panoramic radiograph obtained at eleven years showed no progression compared with the ten-year image (Figure 6 and Figure 7). Subsequent imaging at eleven years of age confirmed persistent short roots of the permanent teeth, with no evidence of continued odontogenesis (Figure 8). These findings suggest the early arrest of root formation rather than progressive external root resorption.

### 2.4. Oral Management and Follow-Up

The patient was enrolled in a long-term preventive oral care program. Professional mechanical tooth cleaning, topical fluoride application, and individualized oral hygiene instruction were provided at approximately monthly intervals. This preventive approach was selected in consideration of the patient’s immunocompromised status and increased susceptibility to infection.

**Figure 9:**
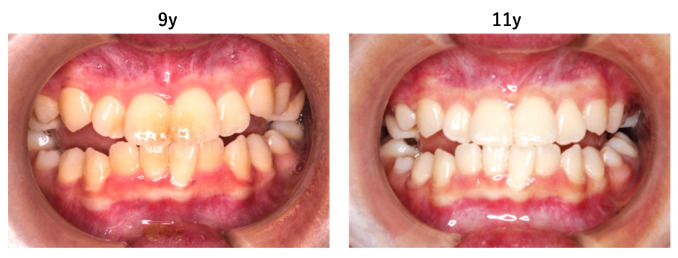
Posterior tooth eruption had not progressed, and occlusion was supported mainly by the anterior teeth, resulting in the marked mobility of the mandibular anterior teeth.

At nine and 11 years of age, the eruption of the posterior teeth had not progressed, and occlusion was primarily supported by the anterior teeth. Consequently, the marked mobility of the mandibular anterior teeth was observed (Figure 9). As the mandibular anterior teeth exhibited mobility and there was a high risk of tooth dislodgement during endotracheal intubation, preventive measures were implemented when surgical procedures were planned. In addition to routine oral care, dental impressions were obtained in advance, and a custom-made mouthpiece was fabricated and applied to prevent accidental tooth loss during intubation.

**Figure 10:**
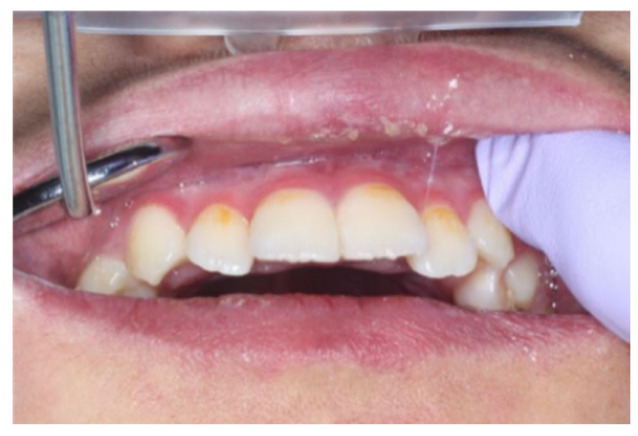
No dental caries were observed in the oral cavity; however, gingivitis and plaque accumulation were present.

At the age of ten years, during evaluation and treatment for multiple brain abscesses, a bacteriological examination of dental plaque was performed to assess the potential oral sources of infection (Figure 10). Supragingival plaque was collected as part of routine clinical sampling and submitted to the institutional microbiology laboratory for culture-based identification according to standard procedures. The analysis detected *Rothia aeria* and *Corynebacterium durum*, organisms that have been associated with opportunistic infections in immunocompromised hosts [25,26,27]. These organisms were identified in oral specimens; microbiological data from the brain abscesses were not available for direct comparison in this report. Following the systemic treatment and improvement of the brain abscesses, repeat bacteriological examination one year later showed a normalization of the oral flora.

**Figure 11:**
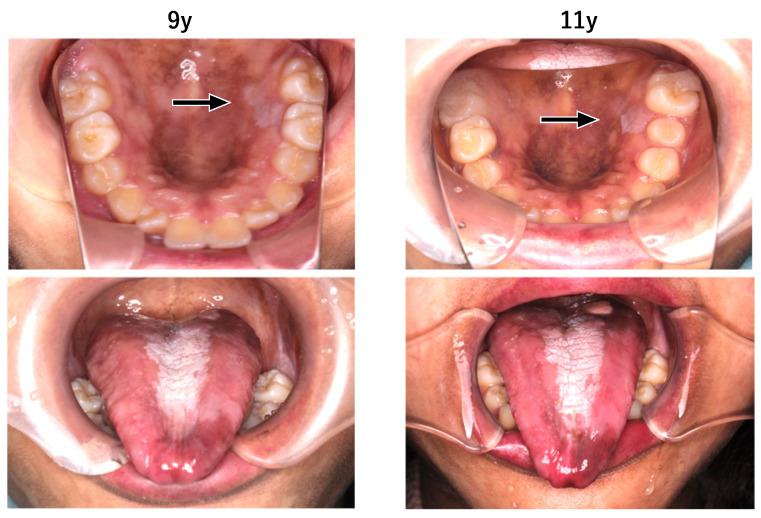
White lesions (arrows) were observed on the palatal aspect of the left maxillary molar region and on the tongue at both nine and 11 years of age. No significant changes were noted over time. Cytological findings of the white lesion on the dorsal surface of the tongue were classified as Class II, indicating no malignant changes.

Leukoplakic lesions were observed on the palatal aspect of the left maxillary molar region and on the tongue. No apparent changes were noted between nine and 11 years of age. (Figure 11) A cytological examination of the white lesion on the dorsal surface of the tongue was performed at the age of eleven years. The lesion was classified as Class II, indicating inflammatory changes without evidence of malignancy. Based on this result and the patient’s hematologic/immunologic vulnerability, we selected continued noninvasive surveillance rather than an immediate biopsy. The lesion was documented clinically (including size and surface characteristics) at each follow-up visit, and a repeat cytology or biopsy was planned if any interval change suggestive of dysplasia or malignancy emerged. Regular clinical surveillance of the oral mucosa was continued.

At the age of thirteen years, the patient underwent a bilateral lung transplantation due to progressive hypoxemia caused by pulmonary arteriovenous malformations. Postoperatively, she was maintained on long-term immunosuppressive therapy with prednisolone, cyclosporine, and mycophenolate mofetil. Although respiratory function improved, progressive renal and hepatic dysfunction developed. She subsequently underwent living-donor liver transplantation but ultimately died of septic shock in November 2024.

## 3. Discussion

Dyskeratosis congenita (DC) is a prototypical telomere biology disorder (TBD) with complex multisystem manifestations and progressive clinical course [1,2,3]. As telomere shortening limits the proliferative capacity of stem and progenitor cells, tissues with high turnover—including the oral mucosa and developing dentition—may be particularly susceptible to developmental disturbances and impaired repair [1,2,3].

### 3.1. Dental Root Development and Telomere Dysfunction

Dental root formation is a highly regulated process that begins after crown completion and continues for several years. It requires the sustained activity of Hertwig’s epithelial root sheath, odontoblast differentiation, and coordinated dentin–cementum deposition. In DC/TBDs, telomere-driven stem-cell exhaustion and impaired tissue regenerative capacity may plausibly disturb these processes and contribute to the developmental short-root phenotypes reported in prior cohorts and case series [3,27,28].

In the present case, serial panoramic radiographs suggested that the root development of several permanent teeth stalled during early childhood, resulting in generalized short roots without radiographic features of inflammatory or orthodontic root resorption. Similar distinctions between developmental short roots and acquired resorption have been emphasized in the dental late-effects literature, where the timing of systemic insults during odontogenesis is a key determinant of root outcomes [17,18,19,20,21,22,23].

### 3.2. Impact of Chemotherapy and Systemic Treatment

In addition to intrinsic telomere dysfunction, exposure to chemotherapy during a critical window of odontogenesis may have contributed to the observed root developmental arrest. Alkylating agents and other cytotoxic regimens administered in early childhood have been associated with dental developmental anomalies, including shortened roots, microdontia, and enamel defects, particularly when exposure occurs before the completion of root formation [17,18,19,20,21,22,23].

Given that HSCT occurred at five years of age in this patient, the conditioning regimen plausibly overlapped with the active root development of multiple permanent teeth. Although the relative contribution of DC biology versus treatment effects cannot be determined in a single case, integrating exposure timing with longitudinal imaging may help clinicians interpret mixed etiologies and anticipate functional risk in similar patients [14,15,16,17,18,19,20].

### 3.3. Oral Health Management in Medically Complex Children

Despite severe systemic disease and long-term immunosuppression, the patient maintained relatively good oral health without caries during her follow-ups. This outcome underscores the value of continuous preventive oral care, including professional cleaning, fluoride exposure, and the reinforcement of home oral hygiene in medically complex children [21].

Preventive strategies in DC/TBDs must be individualized according to hematologic status, mucosal fragility, and planned medical interventions. Standard pediatric oncology-focused dental guidance emphasizes the early identification and elimination of oral infection sources, the minimization of traumatic procedures during immunosuppression, and close coordination with the medical team, principles that are directly applicable to DC patients undergoing HSCT or long-term immunosuppression [27].

The detection of opportunistic bacteria in dental plaque during systemic infection highlights the potential role of the oral cavity as a reservoir for pathogens in immunocompromised hosts. Commensal genera such as *Rothia* and nondiphtheriae *Corynebacterium* species are increasingly recognized as opportunistic pathogens capable of invasive disease in susceptible patients, including bacteremia, endocarditis, and other deep-seated infections reported in the literature [25,26,27]. Although we could not link the oral isolates to the brain abscesses in this case, the finding supports maintaining a low oral microbial burden through meticulous hygiene and professional care as part of infection-prevention strategies [24,25,26,27].

Interdisciplinary collaboration between dental and medical teams is therefore essential in the comprehensive management of patients with DC/TBDs, particularly around HSCT and periods of profound cytopenia or immunosuppression [2,3,24].

### 3.4. Risk of Oral Malignancy and Surveillance

Patients with DC are at a markedly increased risk of squamous cell carcinoma (SCC), particularly in the head and neck region, and this risk may present at younger ages than in the general population [3,16,28,29,30]. Oral leukoplakia is a common mucosal manifestation of DC and warrants careful longitudinal surveillance as a potentially malignant disorder within this high-risk background [3,16,28,29,30].

Mechanistically, critically short telomeres promote genomic instability, impaired DNA damage responses, and clonal selection, which together can facilitate malignant transformation [1,2,3]. In DC, these intrinsic carcinogenic pressures may be compounded by chronic mucosal injury, impaired wound healing, and persistent inflammation [3,14,15,16,28].

In addition to intrinsic telomere-related mechanisms, chronic mucosal injury and impaired tissue repair may further contribute to carcinogenesis in DC. Oral leukoplakia, a common manifestation of DC, is considered a potentially malignant disorder and often arises in the setting of epithelial fragility, chronic inflammation, and defective regenerative capacity. Unlike conventional leukoplakia in the general population, leukoplakic lesions in DC may progress to SCC even in the absence of traditional risk factors such as tobacco or alcohol exposure, underscoring the unique biological vulnerability of these patients.

Iatrogenic factors may also modify cancer risk in DC. HSCT, chemotherapy, and long-term immunosuppression can alter immune surveillance and mucosal biology, potentially contributing to carcinogenesis and emphasizing the need for structured, long-term oral follow-up in survivors [3,28,29,30,31].

From a clinical perspective, the association between DC and oral SCC supports a low threshold for the biopsy of suspicious lesions and regular specialist examinations. Current reviews emphasize vigilant head and neck surveillance in DC/TBDs, especially in patients with leukoplakia or prior HSCT [3,28,29,30,31].

In the present case, tongue leukoplakia was identified during childhood and monitored longitudinally without evidence of malignant transformation during follow-up, illustrating both the feasibility and importance of sustained surveillance in pediatric DC [3,10,11,12,13].

### 3.5. Clinical Implications

This case adds to the limited body of literature describing long-term oral outcomes in pediatric DC. The combination of continuous preventive management and serial imaging provided a clinically useful framework for documenting dental development and anticipating functional risk in a rare, medically complex condition [3,13,24].

Longitudinal imaging-based assessment provides the objective documentation of dental development over time and may help clinicians distinguish developmental disturbances related to systemic disease from treatment-related sequelae, which is particularly valuable where evidence-based dental guidelines are limited [14,15,16,17,18,19,20,21].

Taken together, prior reports suggest that telomere dysfunction in DC/TBDs can contribute to abnormal root development, while systemic therapies administered during early childhood may further exacerbate dental developmental disturbances. The accumulation of similar longitudinal datasets across institutions may ultimately support more standardized recommendations for oral care and surveillance in telomere biology disorders [1,2,3,13,17,18,19,20,21,22,23,24].

### 3.6. Limitations

This report has several limitations inherent to single-case studies. First, causal relationships between telomere dysfunction, chemotherapy exposure, and arrested dental root development cannot be definitively established based on one patient. The observed dental findings were likely multifactorial, and the relative contribution of the underlying telomere biology disorder versus systemic treatment effects remains speculative. Second, quantitative assessments such as three-dimensional imaging or the histological evaluation of dental tissues were not available, limiting the detailed analysis of root morphology and developmental mechanisms. Third, because this patient had severe multisystem disease and underwent multiple organ transplantations, the findings may not be generalizable to all patients with dyskeratosis congenita, particularly those with milder phenotypes. Finally, the long-term functional outcomes into adulthood could not be evaluated due to the patient’s clinical course. Larger case series and longitudinal studies are needed to better clarify the mechanisms and clinical spectrum of dental abnormalities in dyskeratosis congenita.

## 4. Conclusions

We reported a rare pediatric case of dyskeratosis congenita with long-term follow-up focusing on oral functional management and dental development. Serial imaging demonstrated the early cessation of dental root formation, likely associated with the combined effects of telomere dysfunction and chemotherapy administered during early childhood. Continuous preventive oral care and imaging-based monitoring were effective in maintaining oral health and detecting mucosal changes in this medically complex patient. Long-term oral management should be considered an integral component of multidisciplinary care for patients with dyskeratosis congenita.

## Data Availability

The original contributions presented in this study are included in the article. Additional data are not publicly available due to privacy and ethical restrictions.

## References

[1] Kam M.L.W., Nguyen T.T.T., Ngeow J.Y.Y. (2021). Telomere biology disorders. npj Genom. Med..

[2] Niewisch M.R., Savage S.A. (2019). An update on the biology and management of dyskeratosis congenita and related telomere biology disorders. Expert. Rev. Hematol..

[3] Savage S.A. (2022). Dyskeratosis congenita and telomere biology disorders. Hematol. Am. Soc. Hematol. Educ. Program..

[4] Bertuch A.A. (2016). The molecular genetics of the telomere biology disorders. RNA Biol..

[5] Niewisch M.R., Giri N., McReynolds L.J., Alsaggaf R., Bhala S., Alter B.P., Savage S.A. (2022). Disease progression and clinical outcomes in telomere biology disorders. Blood.

[6] Walne A.J., Collopy L., Cardoso S., Ellison A., Plagnol V., Albayrak C., Albayrak D., Kilic S.S., Patıroglu T., Akar H. (2016). Marked overlap of four genetic syndromes with dyskeratosis congenita confounds clinical diagnosis. Haematologica.

[7] Khincha P.P., Dagnall C.L., Hicks B., Jones K., Aviv A., Kimura M., Katki H., Aubert G., Giri N., Alter B.P. (2017). Correlation of leukocyte telomere length measurement methods in patients with dyskeratosis congenita and in their unaffected relatives. Int. J. Mol. Sci..

[8] Higgs C., Crow Y.J., Adams D.M., Chang E., Hayes D., Herbig U., Huang J.N., Himes R., Jajoo K., Johnson F.B. (2019). Understanding the evolving phenotype of vascular complications in telomere biology disorders. Angiogenesis.

[9] Himes R.W., Chiou E.H., Queliza K., Shouval D.S., Somech R., Agarwal S., Jajoo K., Ziegler D.S., Kratz C.P., Huang J. (2021). Gastrointestinal hemorrhage: A manifestation of the telomere biology disorders. J. Pediatr..

[10] Revy P., Kannengiesser C., Fischer A. (2019). Somatic genetic rescue in Mendelian haematopoietic diseases. Nat. Rev. Genet..

[11] Ward S.C., Savage S.A., Giri N., Alter B.P., Rosenberg P.S., Pichard D.C., Cowen E.W. (2018). Beyond the triad: Inheritance, mucocutaneous phenotype, and mortality in a cohort of patients with dyskeratosis congenita. J. Am. Acad. Dermatol..

[12] Atkinson J.C., Harvey K.E., Domingo D.L., Trujillo M.I., Guadagnini J.P., Gollins S., Giri N., Hart T.C., Alter B.P. (2008). Oral and dental phenotype of dyskeratosis congenita. Oral Dis..

[13] Koruyucu M., Barlak P., Seymen F. (2014). Oral and dental findings of dyskeratosis congenita. Case Rep. Dent..

[14] AlSabbagh M.M. (2020). Dyskeratosis congenita: A literature review. J. Dtsch. Dermatol. Ges..

[15] Serindere G. (2018). Oral and dental changes of dyskeratosis congenita. J. Adv. Oral Res..

[16] Noto Z., Tomihara K., Furukawa K., Noguchi M. (2016). Dyskeratosis congenita associated with leukoplakia of the tongue. Int. J. Oral Maxillofac. Surg..

[17] Gallenzi P., Malatesta A., Staderini E., Guglielmi F. (2025). Chemotherapy with Alkylating Agents and Dental Anomalies in Children: A Systematic Review. J. Clin. Med..

[18] Kang C.-M., Hahn S.M., Kim H.S., Lyu C.J., Lee J.-H., Lee J., Han J.W. (2017). Clinical risk factors influencing dental developmental disturbances in childhood cancer survivors. Cancer Res. Treat..

[19] Proc P., Szczepańska J., Skiba A., Zubowska M., Fendler W., Młynarski W. (2016). Dental anomalies as late adverse effect among young children treated for cancer. Cancer Res. Treat..

[20] Halperson E., Matalon V., Goldstein G., Spilberg S.S., Herzog K., Fux-Noy A., Shmueli A., Ram D., Moskovitz M. (2022). The prevalence of dental developmental anomalies among childhood cancer survivors according to type of anticancer treatment. Sci. Rep..

[21] Seremidi K., Kavvadia K., Kattamis A., Polychronopoulou A. (2021). Dental late effects of antineoplastic treatment on childhood cancer survivors: Radiographic findings. Int. J. Paediatr. Dent..

[22] Ruzman L., Zulijani A., Skrinjaric T., Buljan D., Giljevic J.S., Cace I.B., Milardovic A. (2026). Late Oral Complications in Childhood Cancer Survivors: Implications for Pediatric Dentistry and Survivorship Care. Children.

[23] Seremidi K., Gizani S., Anderson M., Dahllöf G., Barr-Agholme M., Parekh S., Tsilingaridis G., European Academy of Paediatric Dentistry (EAPD) (2025). Best clinical practise guidance for oral health care management of long-term childhood cancer survivors (CCS): An EAPD policy document. Eur. Arch. Paediatr. Dent..

[24] American Academy of Pediatric Dentistry (2025). Dental Management of Pediatric Patients Receiving Chemotherapy, Hematopoietic Cell Transplantation, and/or Radiation Therapy; The Reference Manual of Pediatric Dentistry.

[25] Fatahi-Bafghi M. (2021). Characterization of the Rothia spp. and their role in human clinical infections. Infect. Genet. Evol..

[26] McMullen A.R., Anderson N., Wallace M.A., Shupe A., Burnham C.A. (2017). When good bugs go bad: Epidemiology and antimicrobial resistance profiles of Corynebacterium striatum, an emerging multidrug-resistant, opportunistic pathogen. Antimicrob. Agents Chemother..

[27] Mitchell B.I., Markantonis J.E. (2025). An underestimated pathogen: *Corynebacterium* species. J. Clin. Microbiol..

[28] Trott K.E., Briddell J.W., Corao-Uribe D., Powell J., Seecof O.M., Levy C., Miller E.G., Shah U.K. (2019). Dyskeratosis Congenita and Oral Cavity Squamous Cell Carcinoma: Report of a Case and Literature Review. J. Pediatr. Hematol. Oncol..

[29] Liu A.Q., Deane E.C., Prisman E., Durham J.S. (2022). Dyskeratosis Congenita and Squamous Cell Cancer of the Head and Neck: A Case Report and Systematic Review. Ann. Otol. Rhinol. Laryngol..

[30] Alter B.P., Giri N., Savage S.A., Rosenberg P.S. (2009). Cancer in dyskeratosis congenita. Blood.

[31] Mawardi H., Hashmi S.K., Elad S., Aljurf M., Treister N. (2019). Chronic graft-versus-host disease: Current management paradigm and future perspectives. Oral. Dis..

